# Survival rates and worker compensation expenses in a national cohort of Mexican workers with permanent occupational disability caused by diabetes

**DOI:** 10.1186/s12889-016-3598-4

**Published:** 2016-09-01

**Authors:** Iván de Jesús Ascencio-Montiel, Jesús Kumate-Rodríguez, Víctor Hugo Borja-Aburto, José Esteban Fernández-Garate, Selene Konik-Comonfort, Oliver Macías-Pérez, Ángel Campos-Hernández, Héctor Rodríguez-Vázquez, Verónica Miriam López-Roldán, Edgar Jesús Zitle-García, María del Carmen Solís-Cruz, Ismael Velázquez-Ramírez, Miriam Aguilar-Jiménez, Leonel Villa-Caballero, Nelly Cisneros-González

**Affiliations:** 1 Epidemiological Surveillance Coordination, Mexican Institute of Social Security, Mier y Pesado 120, Col. del Valle, Benito Juárez, 03100 Mexico City, Mexico; 2 Fundación IMSS, A.C., Av. Paseo de la Reforma 476, Col. Juárez, Cuauhtémoc, 06600 Mexico City, Mexico; 3 Primary Health Care Unit, Mexican Institute of Social Security, Hamburgo 18, Col. Juárez, Cuauhtémoc, 06600 Mexico City, Mexico; 4 Division of Technical Studies, Coordination of Economic Benefits, Mexican Institute of Social Security, Toledo 21, Col. Juárez, Cuauhtémoc, 06600 Mexico City, Mexico; 5 Health Information Division, Mexican Institute of Social Security, Durango 289, Col. Roma Norte, Cuauhtémoc, 06700 Mexico City, Mexico; 6 Division of Information Services for Economic and Social Benefits, Mexican Institute of Social Security, Tokio 80, Col. Juárez, Cuauhtémoc, 06600 Mexico City, Mexico; 7 Division of Rehabilitation, Mexican Institute of Social Security, Durango 289, Col. Roma Norte, Cuauhtémoc, 06700 Mexico City, Mexico; 8 Occupational Health Coordination, Mexican Institute of Social Security, Av. Cuauhtémoc 330, Col. Doctores, Cuauhtémoc, 06720 Mexico City, Mexico; 9 University of California, San Diego Extension, 8950 Villa La Jolla Drive, Suite C-215, La Jolla, California 92093-0170 USA

**Keywords:** Diabetes mellitus, Survival, Disability insurance

## Abstract

**Background:**

Permanent occupational disability is one of the most severe consequences of diabetes that impedes the performance of usual working activities among economically active individuals. Survival rates and worker compensation expenses have not previously been examined among Mexican workers. We aimed to describe the worker compensation expenses derived from pension payments and also to examine the survival rates and characteristics associated with all-cause mortality, in a cohort of 34,014 Mexican workers with permanent occupational disability caused by diabetes during the years 2000–2013 at the Mexican Institute of Social Security.

**Methods:**

A cross-sectional analysis study was conducted using national administrative records data from the entire country, regarding permanent occupational disability medical certification, pension payment and vital status. Survival rates were estimated using the Kaplan–Meier method. Multivariate Cox proportional hazard model was used to estimate adjusted hazard ratios (HR) and 95 % confidence intervals (95 % CI) in order to assess the cohort characteristics and all-cause mortality risk. Total expenses derived from pension payments for the period were accounted for in U.S. dollars (USD, 2013).

**Results:**

There were 12,917 deaths in 142,725.1 person-years. Median survival time was 7.26 years. After multivariate adjusted analysis, males (HR, 1.39; 95 % CI, 1.29–1.50), agricultural, forestry, and fishery workers (HR, 1.41; 95 % CI, 1.15–1.73) and renal complications (HR, 3.49; 95 % CI, 3.18–3.83) had the highest association with all-cause mortality. The all-period expenses derived from pension payments amounted to $777.78 million USD (2013), and showed a sustained increment: from $58.28 million USD in 2000 to $111.62 million USD in 2013 (percentage increase of 91.5 %).

**Conclusions:**

Mexican workers with permanent occupational disability caused by diabetes had a median survival of 7.26 years, and those with renal complications showed the lowest survival in the cohort. Expenses derived from pension payments amounted to $ 777 million USD and showed an important increase from 2000 to 2013.

## Background

Diabetes is a growing worldwide public health problem that affects the productive-age population [1], confers an increased risk of disability [2], and places a considerable economic burden on society [3, 4].

In relation with work, it has been shown that subjects with diabetes had higher rates of permanent and temporary work disability, early retirement and premature death in comparison with subjects without diabetes [2, 5, 6]. Permanent occupational disability is one of the most severe consequences of diabetes that impedes the performance of regular working activities among economically active individuals and has a deleterious effect at the societal level. This condition has a high economic cost derived from poor worker productivity life, wages loss and medical benefits from the worker’s compensation insurance [7].

It is known that Diabetes affects predominately low and middle-income nations, including Latin American Countries, where diabetes imposes a high economic burden that increased to $65,216 million U.S. dollars (USD) in 2000 [8]. Mexico has one of the largest populations in the region and one of the highest prevalence of diabetes too [9].

The Mexican Institute of Social Security (IMSS) is the main Health Institution in Mexico and the largest Latin American social security system that provides social services, economic assistance, and health care to ~71 million currently affiliated Mexican citizens, 16.5 million workers of the formal sector and their families.

The IMSS provides services for almost 50 % of the Mexican population and, diabetes constitutes the main cause of death, permanent occupational disability and expenses for chronic diseases at this institution [10]. The total cost for diabetes care at the IMSS was estimated at 909.6 million USD in 2011 [11]. Using a lost output approach, the indirect cost of permanent disability amounted to ~50 % of the total cost (471.9 million USD).

Given that survival and characteristics associated with mortality in patients with permanent occupational disability caused by diabetes has been insufficiently explored, and that the expenses derived for pension payments by the IMSS have not been directly quantified in the past, we aimed the following: 1) to describe the survival rates, 2) to examine the characteristics associated with all-cause mortality and 3) to assess the expenses derived from pension payments, in a cross sectional analysis of a retrospective cohort of 34,014 Mexican workers with permanent occupational disability caused by diabetes during the years 2000–2013.

We hypothesize that, given the severity of the disease, workers with permanent occupational disability caused by diabetes could have a short survival and that the expenses derived of pension payments have increased significantly for that period.

## Methods

### Study design and population

A cross-sectional analysis was conducted with data from the IMSS administrative registers of the whole country, creating a cohort comprised of workers affiliated with the IMSS that fulfill three criteria: 1) to have a permanent occupational disability caused by diabetes, 2) to have an approved compensation claim for disability and 3) to receive a pension payment during the period 2000–2013.

Subjects were identified from three administrative records: 1) the permanent occupational disability database, 2) the pension payment system and 3) the IMSS death registration system. The first database maintains the permanent occupational disability medical certificate records, whereas the pension payment system stores the pension payment records, attendance of the worker at the IMSS facilities and the discharge (stop of the payment) from the pension system due to the worker’s death as well. The three mentioned databases provided information from the 32 states that encompass the Mexican territory.

### Measurements

Data for each worker including sex, age at time of permanent occupational disability, occupation, main complication of diabetes, death, base salary contribution and accumulated pension payment amount were acquired. Occupation was categorized into the nine major groups of the International Standard Classification of Occupations (ISCO-88) [12].

Diabetes complications were recorded for each worker corresponding to the main complication related to the permanent occupational disability. This diabetes complication was classified into one of five categories according to the International Classification of Diseases, 10th revision (ICD-10) [13]: renal, ophthalmic, neurological, peripheral circulatory, or multiple. Workers with unavailable or unspecified information on the main diabetes complication or occupation were assigned to separate unspecified groups.

### Expense assessment

Base salary contribution and expenses derived from the pension payment were obtained from the previously mentioned payment system. The base salary contribution corresponded to the worker’s daily salary at registration at the IMSS, and was used to estimate the lifetime pension amount. Expenses derived from pension payments were obtained by adding the pension payroll of each subject from the time of enrollment until death or December 2013.

The two above-mentioned expense measures were accounted for in inflation-adjusted 2013 Mexican pesos (using the average Consumer Price Index observed for the period 2000–2013). Results were expressed in USD (1 USD = 12.77 Mexican pesos) corresponding to the annual mean of 2013.

### Follow-up of vital status

The vital status information from each worker was obtained from the pension payment system, which accumulates the registers of the pensioned worker’s attendance at the IMSS facilities every 6 months, and the discharge from the pension system because of death. This last condition was obtained through the monthly status check within the IMSS death registration system.

Person-years of follow-up began January 1, 2000 and ended at death or at the end of follow-up (December 31, 2013), whichever came first.

### Statistical analysis

Survival rates were calculated using the Kaplan–Meier method. Survival curves were constructed for each variable of this cohort including sex, age at permanent occupational disability, occupation, main complication of diabetes and base salary contribution.

All-cause mortality rates were analyzed across the categories for each variable of the cohort. Multivariable cox proportional hazard model was used to estimate adjusted hazard ratios (HR) and 95 % confidence intervals (95 % CI) in order to assess the association between the characteristics of the cohort and all-cause mortality.

The number of subjects who received pension payments and the total amount of expenses derived from pension payments were accounted for each year of the period.

Statistical analysis was carried out using R software v.3.0.1 with the following packages: survival, epicalc, and epitools.

## Results

### Subjects

A total of 34,014 Mexican workers affiliated with the IMSS with permanent occupational diabetes-caused disability from 2000 to 2013 were included in this cohort. The mean age at permanent occupational disability was 51.6 years old. The majority of the workers were male. The most frequent main complication of diabetes causing the permanent occupational disability was ophthalmic related. Table 1 shows the full characteristics of this cohort.

**Table 1:** Characteristics of the workers with permanent occupational disability^a^

Characteristic	Total cohort
No. of workers	34,014
Sex (%)	
Male	84.3
Female	15.7
Age at permanent occupational disability, years (mean ± SD)	51.6 ± 7.6
Age at permanent occupational disability (%)	
<40 years	7.0
40–59 years	83.9
≥60 years	9.1
Occupation group (%)	
Managers	3.4
Professionals	4.0
Technicians and associate professionals	4.4
Clerks	11.5
Service and sales	13.2
Agricultural, forestry, and fishery	2.7
Craft and related trades	13.3
Plant, machine operators and assemblers	24.7
Basic-level occupations	22.5
No occupation specified	0.3
Base salary contribution group (%)^b^	
<10 USD/day	33.6
10–30 USD/day	23.9
>30 USD/day	6.4
Main complication of diabetes (%)^c^	
Renal	12.9
Ophthalmic	40.6
Neurological	11.9
Peripheral circulatory disorders	15.6
Multiple	17.1
No specified complication	1.9

Data are presented as mean ± SD or percentages

^a^The study included Mexican workers with an approved compensation claim for permanent occupational disability caused by diabetes and who received a pension payment during the period 2000–2013

^b^Corresponds to the daily salary at which a worker was registered at the Mexican Institute of Social Security and was used to calculate the lifetime individual pension amount

^c^Denotes the main complication that caused the permanent occupational disability status

USD indicates U.S. dollars

### Survival

**Fig. 1:**
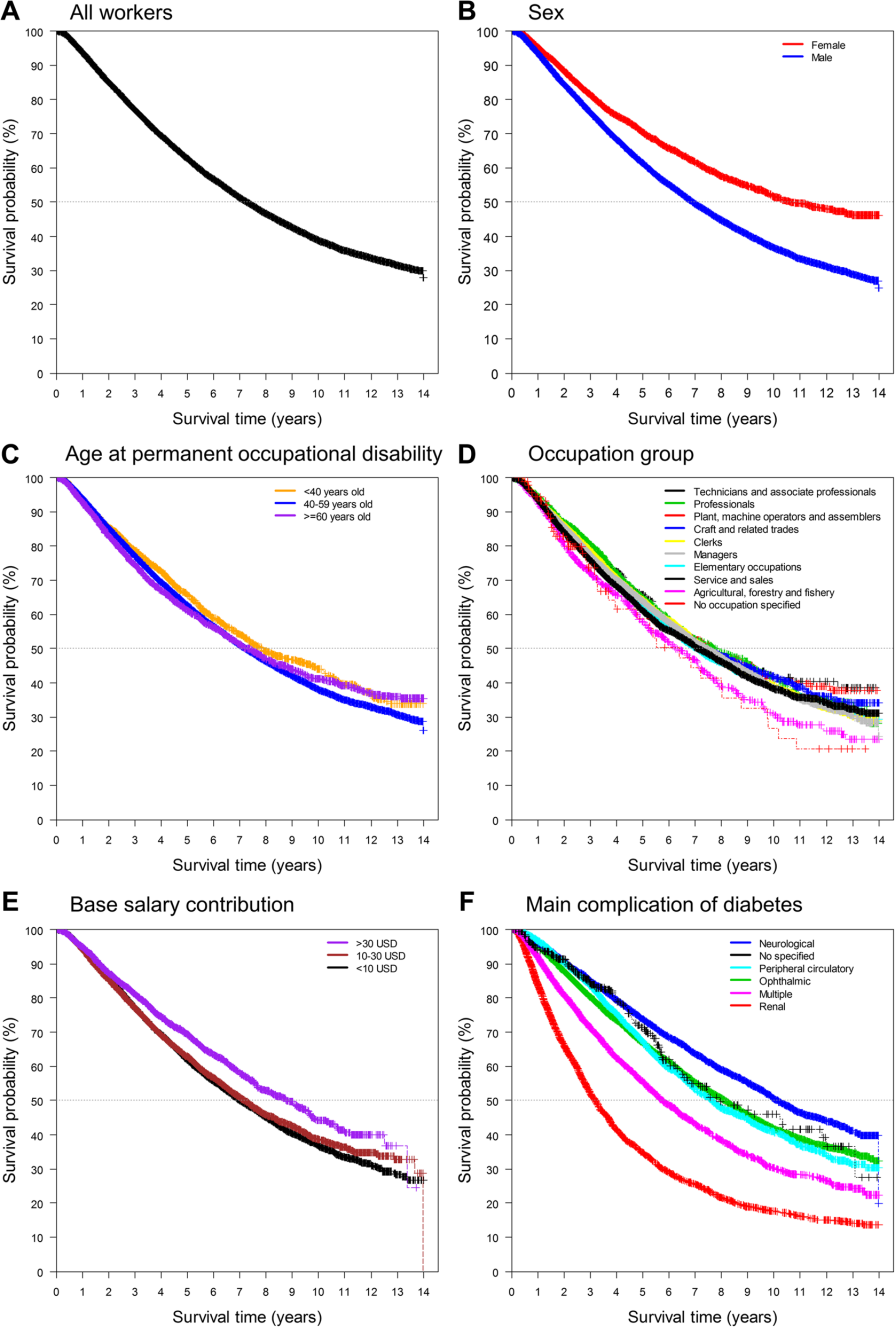
Survival according to workers’ characteristics. **a** Kaplan–Meier estimates of overall survival among Mexican workers with permanent occupational disability caused by diabetes. **b** Kaplan–Meier estimates of overall survival in males vs. females (*P* = 2.26 × 10^−43^). **c**–**f** The same estimates across ages of permanent occupational disability groups (*P* = 0.029), occupation groups (*P* = 0.001), base salary contribution groups (*P* = 1.10 × 10^−10^) and diabetes main complication groups (*P* < 2.95 × 10^−308^) are shown, respectively

There were 12,917 deaths from all causes involving 142,725.1 person-years with a follow-up mean of 3.26 years and a maximum follow-up of 13.9 years. 37.9 % of workers included in the study died during this follow-up period. Median survival time was 7.26 years. Mean (±SD) age at death was 55.5 ± 8.0 years. Higher death rates were observed among older male workers, workers with a lower base salary contribution, those with renal complications, and among those working in the agricultural, forestry, and fishery industries (Fig. 1 and Table 2).

**Table 2:** Hazard ratios for all-cause mortality^a^

Characteristic	No. of workers	No. of deaths	Person-years	All-cause mortality rate^d^	HR (95 % CI)	Adjusted HR (95 % CI)
Sex						
Female	5,339	1,526	23,271.3	65.6	1.00	1.00
Male	28,675	11,391	119,453.9	95.4	1.45 (1.38–1.53)	1.39 (1.29–1.50)
Age at permanent occupational disability						
<40 years	2,384	842	10,151.2	82.9	1.00	1.00
40–59 years	28,551	10,811	118,719.3	91.1	1.10 (1.02–1.18)	1.18 (1.06–1.30)
≥60 years	3,079	1,264	13,854.7	91.2	1.10 (1.01–1.20)	1.15 (1.01–1.31)
Occupation group						
Managers	1,167	290	3,571.0	81.2	1.00	1.00
Technicians and associate professionals	1,485	499	6,001.7	83.1	1.02 (0.89–1.18)	1.08 (0.89–1.30)
Professionals	1,365	428	4,902.2	87.3	1.08 (0.93–1.25)	1.20 (0.99–1.45)
Clerks	3,913	1,267	14,411.0	87.9	1.08 (0.95–1.23)	1.20 (1.02–1.42)
Craft and related trades	4,532	1,798	20,264.5	88.7	1.09 (0.97–1.24)	1.09 (0.93–1.29)
Plant, machine operators and assemblers	8,396	3,284	36,652.7	89.6	1.10 (0.98–1.24)	1.11 (0.95–1.29)
Service and sales	4,490	1,862	20,183.6	92.3	1.14 (1.00–1.29)	1.16 (0.99–1.36)
Basic-level occupations	7,655	3,008	32,354.9	93.0	1.14 (1.01–1.29)	1.22 (1.04–1.42)
Agricultural, forestry, and fishery	916	443	4,044.2	109.5	1.35 (1.16–1.56)	1.41 (1.15–1.73)
No occupation specified	95	38	339.3	112.0	1.38 (0.98–1.93)	1.60 (0.93–2.76)
Base salary contribution (per day)^b^						
>30 USD	2,166	432	6,388.4	67.6	1.00	1.00
10–30 USD	8,095	2,133	25,796.4	82.7	1.22 (1.10–1.36)	1.23 (1.11–1.37)
<10 USD	11,500	4,182	44,827.9	93.3	1.38 (1.25–1.52)	1.36 (1.22–1.51)
Main complication of diabetes^c^						
Neurological	4,045	1,505	23,753.7	63.4	1.00	1.00
Not specified	643	168	2,359.0	71.2	1.12 (0.96–1.32)	1.25 (1.01–1.54)
Peripheral circulatory disorders	5,310	1,690	21,864.6	77.3	1.22 (1.14–1.31)	1.21 (1.09–1.34)
Ophthalmic	13,830	4,948	61,973.0	79.8	1.26 (1.19–1.33)	1.30 (1.19–1.42)
Multiple	5,810	2,021	19,350.0	104.4	1.65 (1.54–1.76)	1.79 (1.62–1.97)
Renal	4,376	2,585	13,424.8	192.6	3.04 (2.85–3.24)	3.49 (3.18–3.83)

^a^Hazard ratios were adjusted using a Cox proportional hazard model

^b^Corresponds to the daily salary at which a worker was registered at the Mexican Institute of Social Security. This was used to calculate the lifetime individual pension amount

^c^Denotes the main complication that caused the permanent occupational disability state

^d^All-cause mortality rates are expressed as number of deaths/1,000 person-years

USD indicates U.S. dollars; HR indicates hazard ratio and CI indicates confidence interval

### Characteristics associated with all-cause mortality

After multivariate adjustment, the characteristics associated with significantly elevated all-cause mortality were having renal complications (HR, 3.49; 95 % CI, 3.18–3.83), having multiple complications (HR, 1.79; 95 % CI, 1.62–1.97), belonging to the agricultural, forestry, or fishery workers group (HR, 1.41; 95 % CI, 1.15–1.73), male gender (HR, 1.39; 95 % CI, 1.29–1.50) and having a base salary contribution <10 USD/day (HR 1.36; 95 % CI, 1.22–1.51).

### Worker compensation expenses derived from pension payments

A sustained increase in the number of subjects who received worker’s compensation pension payments was observed (from 1018 in 2000 to 18,818 individuals in 2013).

**Fig. 2:**
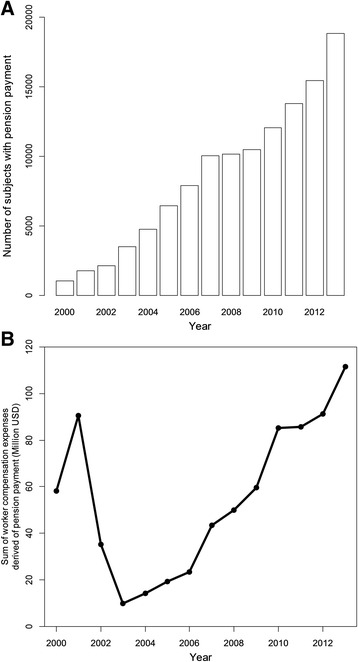
Worker compensation expenses derived from pension payments. **a** Number of subjects who received pension payments in each year from 2000 to 2013 are shown. **b** Total amount of worker compensation expenses derived from pension payments (million U.S. dollars) in each year of the same period are shown

The all-period work compensation expenses derived from pension payments amounted to $777.78 million USD: from $58.28 million USD in 2000 to $111.62 million USD in 2013, with a percentage increase of 91.5 %. A J-shaped expense curve was observed due to a 2001 peak corresponding to internal policies intended to boost the pension regime reform followed by a decrease in 2002 caused by a substitution effect associated as well with internal policies. Subsequently (from 2003 to 2013), a sustained increase in worker’s compensation pension payment expenses was clearly observed: from $9.8 million USD in 2003 to $111.6 million USD in 2013 (percentage increase of 1032.6 %) (Fig. 2a). This expenses increment was correlated with the increase in the number of subjects with pension payments (Fig. 2b).

## Discussion

Diabetes represents a worldwide health problem predominantly affecting both developed and developing nations. It is estimated that 80 % of deaths attributable to diabetes occur in low- and middle-income countries [14]. Permanent occupational disability caused by diabetes is one of the most severe consequences of diabetes, thus, an important economic burden must be born by social health systems including the IMSS, a provider of medical services to approximately 71 million Mexicans.

The study subjects represent a subset of the total number of Mexican workers who participate in the formal labor sector, i.e., those employed with regular wages and hours and with employment rights and tax payments. This formal worker sector represents ~40 % of total workers in Mexico [15].

Mexican workers with permanent occupational disability caused by diabetes had a median survival time of 7.26 years. Mean of age at permanent occupational disability was 51.6 years old. The worker compensation expenses derived from pension payments increased to $777 million USD during the 2000–2013 period.

Our findings regarding average age at death (55.5 years old) and survival curves were similar to what was reported in the Herquelot study, with 506 employees with diabetes [5]; however, the survival curve shown in our study had a larger sample size, providing a significantly more statistical power.

It is known that subjects with end-stage renal disease [16] and with low socioeconomic status [17] had higher mortality rates. Our study also revealed that male workers with renal complications, those with a base salary contribution <10 USD/day, and agricultural, forestry, and fishery workers, experienced the highest all-cause mortality rates.

Employer-paid nonmedical costs of diabetes have been little explored in the literature in the past. Kamal-Bahl [18] estimated disability insurance costs of $31,671 per subject with end-stage renal disease using a hypothetical cohort of 10,000 individuals. In our study, we used a direct quantification of worker compensation expenses derived from pension payments, revealing a total of $777 million USD for 34,014 subjects, with a mean cost of $22,866 USD per subject.

Previous reports have estimated that indirect costs of permanent disability caused by diabetes at the IMSS were 100.8 million USD in 2005, and 471.9 million USD in 2012 [11, 19]. This cost estimate differs from the one in our study, since it uses a lost output approach, where the indirect cost of permanent disability years is estimated and the productive life lost is multiplied by the per capita Gross National Product (GNP). Despite this methodological difference, a similar 4.5-fold cost increase from 19.3 million USD in 2005 to 85.8 million USD in 2012 was observed in our study during the period 2005–2015, reflecting the increasing costs of diabetes at the IMSS.

An evident important increase was observed in the number of subjects who received worker’s compensation pension payments and in the worker’s compensation pension payment expenses and reflects the increase in the diabetes epidemic in Mexico.

The J-shaped expense curve observed was due to a 2001 peak corresponding to internal policies intended to boost the pension regime reform. The reform consists of lifetime annuities funded mainly by the IMSS, but with payments being managed by an insurance company.

With regard to diabetes complications causing permanent occupational disability, ophthalmic disease was the most frequent complication of diabetes.

It is well established that ophthalmological disease is the leading cause of blindness, among patients with diabetes, resulting in significant disability [20] and loss of productive working years. Among the workers in our study, 13.4 years of productive life lost per worker were found, using 65 years old as a reference for age at retirement.

Similar findings were reported other researchers in university employees in Argentina during 1984–1986, who reported retinal lesions as the most frequent cause of diabetes-induced disability and average work production loss per patient of 11 years [21].

Among the limitations of our study was the fact that since we used large administrative databases at the IMSS, the ability to obtain further detailed clinical information like the age of onset diabetes, duration of diabetes, obesity, nutrition status, metabolic parameters, mental comorbidities, medical treatment and therapeutic adherence data, making it difficult to distinguish between type 1 and type 2 diabetes.

Despite these limitations, our study was the first analysis of a national cohort, with a large sample size (*n* = 34,014), that included participants of all the Mexican regions.

This is one of the unique reports where we could obtain a prospective follow-up of more of 90 % of the subjects, and that includes the direct quantification method of worker’s compensation expenses derived from annual pension payments.

To our knowledge, this is the first large cross-sectional analysis with information of the entire country, that examines a national worker population with permanent occupational disability caused by diabetes in Mexico. Studies seeking more detailed information regarding clinical and metabolic conditions, diabetes evolution, therapeutic matters and other indirect costs are sorely needed to improve the knowledge among these workers to reduce the mortality and the economic burden of this diabetes epidemic in this developing country.

Given the results of this and other similar studies, it is expected that diabetes will continue to impact negatively health care systems in both developed and developing nations around the world. The high rates of premature disability and early death (21) observed among economically active populations, as well as the productive life lost (5) and cost related with permanent disability (11, 12), create a worrisome scenario where more aggressive preventive medicine and early public health interventions are sorely needed.

Thus, emerging countries such as Mexico and others from the Latin American region should emphasize more government-funded and private interventions to improve an early diagnosis of the disease in addition of increasing the diabetes awareness at the general population level. It is also a clear priority to enhance the number of effective medical strategies to reduce the high prevalence of long-term diabetes complications which have an elevated cost and deleterious effects at the economies of vulnerable societies.

## Conclusions

Mexican workers with permanent occupational disability caused by diabetes had a median survival of 7.26 years, and those with renal complications showed the lowest survival in the cohort. Expenses derived from pension payments amounted to $ 777 million USD: from $58.28 million USD in 2000 to $111.62 million USD in 2013, with a percentage increase of 91.5 %.

Our study results provide significant evidence of a worrisome situation regarding diabetes on the workforce in Mexico. The increase in worker compensation expenses reflects a negative progression of diabetes in the IMSS, the largest healthcare system of the country.

Higher priority should be given to more comprehensive preventive medicine interventions and appropriate medical control, to stop the growth of this diabetes epidemic.

## Abbreviations

CI: Confidence intervals
GNP: Gross national product
HR: Hazard ratios
ICD-10: International classification of diseases, 10th revision
IMSS: Mexican Institute of Social Security
ISCO-88: International standard classification of occupations
USD: U.S. dollars
